# Prophylactic potential of honey and Nigella *sativa* L. against hospital and community-based SARS-CoV-2 spread: a structured summary of a study protocol for a randomised controlled trial

**DOI:** 10.1186/s13063-021-05510-3

**Published:** 2021-09-15

**Authors:** Sohaib Ashraf, Shoaib Ashraf, Rutaba Akmal, Moneeb Ashraf, Larab Kalsoom, Aadil Maqsood, Muhammad Ahmad Imran, Iqra Farooq, Sidra Ashraf, Uzma Nasim Siddiqui, Muhammad Ghufran, Muhammad Kiwan Akram, Nighat Majeed, Sundas Rafique, Zaigham Habib, Muhammad Sarmad Shahab, Adeen Akmal, Zeeshan Shaukat, Zain ul Abdin, Ayesha Khaqan, Shahroze Arshad, Muhammad Abdul Rehman Virk, Mehak Gul, Abeer bin Awais, Muhammad Hassan, Noman Khalid, Qurrat Ul Ain Iqbal, Tausif Ahmad, Muaaz Akram, Ameer Muhammad, Musa Khalil, Aneeq Aslam, Muhammad Umer, Syed Sami Hussain Sherazi, Zartasha Safdar, Sohail Ahmad, Muhammad Bilal, Muhammad Nauman Zahid, Abdulrahman E. Koshak, Abubakar Hilal, Ahmad Azam Malik, Usman Iqbal, Atif Amin Baig, Yaser Masuod Alahmadi, Ayesha Humayun, Amber Malik, Ali Ahmad, Muhammad Ashraf, Qazi Abdul Saboor, Mateen Izhar, DOCTORS LOUNGE consortium, Arz Muhammad, Kanwal Hayat, Ghazala Amjad, Misbah Kousar, Umair Hafeez, Tayyab Mughal, Tayyaba Muzafar, Sibgha Zulfiqar, Saadia Shahzad Alam, Muhammad Imran Anwar, Amber Malik, Talha Mahmud, Ali Arshad, Khawar Nawaz, Muhammad Ismail Khalid Yousaf

**Affiliations:** 1 Department of Cardiology, Shaikh Zayed Post-Graduate Medical Institute, Lahore, Pakistan; 2 Wellman Center for Photomedicine, Massachusetts General Hospital, Harvard Medical School, Boston, USA; 3 Department of Pathobiology, Riphah International, Lahore, Pakistan; 4 Department of Internal Medicine, Sahara Medical College, Narowal, Pakistan; 5 Department of Pharmacology, Mayo Hospital, Kingedward Medical University, Lahore, Pakistan; 6 Department of Medicine, Services Institute of Medical Sciences, Lahore, Pakistan; 7 Department of Pulmonary & Critical Care Medicine, University of Toledo Medical Center, Toledo, Ohio USA; 8 Department of Microbiology, Shaikh Zayed Post-Graduate Medical Institute, Lahore, Pakistan; 9 Department of Paediatric Surgery, Children Hospital, Lahore, Pakistan; 10 Institute of Biochemistry and Biotechnology, University of Veterinary and Animal Sciences, Lahore, Pakistan; 11 Department of Medicine, Shaikh Zayed Post-Graduate Medical Institute, Lahore, Pakistan; 12 ESACHS (Empresa de Servicio Externo de la Asociación Chilena de Seguridad), Punta Arenas, Chile; 13 Department of Animal Nutrition, University of Veterinary and Animal Sciences, Lahore, Pakistan; 14 Department of Internal Medicine, Services Institute of Medical Sciences, Lahore, Pakistan; 15 Department of Radiotherapy, Mayo Hospital, King Edward Medical University, Lahore, Pakistan; 16 Department of Orthopedics, Shaikh Zayed Post-Graduate Medical Institute, Lahore, Pakistan; 17 Department of Internal Medicine, Allied Hospital, Faisalabad Medical University, Faisalabad, Pakistan; 18 Department of Surgery, University of Veterinary and Animal Sciences, Lahore, Pakistan; 19 Department of Gastroenterology, Shaikh Zayed Post-Graduate Medical Institute, Lahore, Pakistan; 20 Department of Pharmacology and Toxicology, University of Veterinary and Animal Sciences, Lahore, Pakistan; 21 Department of Department of Poultry Production, University of Veterinary and Animal Sciences, Lahore, Pakistan; 22 Department of Animal Science, McGill University, Sainte-Anne-de-Bellevue, Quebec Canada; 23 Department of Biology, college of Science, University of Bahrain, Sakhir, Kingdom of Bahrain; 24 Department of Natural Products and Alternative Medicine, Faculty of Pharmacy, King Abdulaziz University, Jeddah, Saudi Arabia; 25 Department of Family and Community Medicine, Rabigh Faculty of Medicine, King Abdulaziz University, Jeddah, Saudi Arabia; 26 Global Health and Development Department, College of Public Health, Taipei Medical University, Taipei, Taiwan; 27 Unit of Biochemistry, Faculty of Medicine, University Sultan Zainal Abidin, Terengganu, Malaysia; 28 Clinical and Hospital Pharmacy Department, College of Pharmacy, Taibah University Medina, Medina, Kingdom of Saudi Arabia; 29 Department of Community Medicine and Public Health|, Shaikh Zayed Post-Graduate Medical Complex, Lahore, Pakistan; 30 Department of Microbiology, Infectiology and Immunology, Centre Hospitalier Universitaire (CHU) Sainte Justin/University of Montreal, Montreal, Canada

**Keywords:** Honey, Nigella Sativa, Prophetic Medicine, Pakistan, COVID-19, Randomised controlled trial, Protocol

## Abstract

**Objectives:**

Considering the therapeutic potential of honey and Nigella sativa (HNS) in coronavirus disease 2019 (COVID-19) patients, the objective of the study is defined to evaluate the prophylactic role of HNS.

**Trial design:**

The study is a randomized, placebo-controlled, adaptive clinical trial with parallel group design, superiority framework with an allocation ratio of 1:1 among experimental (HNS) and placebo group. An interim analysis will be done when half of the patients have been recruited to evaluate the need to adapt sample size, efficacy, and futility of the trial.

**Participants:**

All asymptomatic patients with hospital or community based COVID-19 exposure will be screened if they have had 4 days exposure to a confirmed case. Non-pregnant adults with significant exposure level will be enrolled in the study

- High-risk exposure (<6 feet distance for >10min without face protection)
- Moderate exposure (<6 feet distance for >10min with face protection)

Subjects with acute or chronic infection, COVID-19 vaccinated, and allergy to HNS will be excluded from the study.

Recruitment will be done at Shaikh Zayed Post-Graduate Medical Institute, Ali Clinic and Doctors Lounge in Lahore (Pakistan).

**Intervention and comparator:**

In this clinical study, patients will receive either raw natural honey (0.5 g) and encapsulated organic Nigella sativa seeds (40 mg) per kg body weight per day or empty capsule with and 30 ml of 5% dextrose water as a placebo for 14 days. Both the natural products will be certified for standardization by Government College University (Botany department). Furthermore, each patient will be given standard care therapy according to version 3.0 of the COVID-19 clinical management guidelines by the Ministry of National Health Services of Pakistan.

**Main outcomes:**

Primary outcome will be Incidence of COVID-19 cases within 14 days of randomisation. Secondary endpoints include incidence of COVID-19-related symptoms, hospitalizations, and deaths along with the severity of COVID-19-related symptoms till 14^th^ day of randomization.

**Randomisation:**

Participants will be randomized into experimental and control groups (1:1 allocation ratio) via the lottery method. There will be stratification based on high risk and moderate risk exposure.

**Blinding (masking):**

Quadruple blinding will be ensured for the participants, care providers and outcome accessors. Data analysts will also be blinded to avoid conflict of interest. Site principal investigator will be responsible for ensuring masking.

**Numbers to be randomised (sample size):**

1000 participants will be enrolled in the study with 1:1 allocation.

**Trial Status:**

The final protocol version 1.4 was approved by institutional review board of Shaikh Zayed Post-Graduate Medical Complex on February 15, 2021. The trial recruitment was started on March 05, 2021, with a trial completion date of February 15, 2022.

**Trial registration:**

Clinical trial was registered on February 23, 2021, www.clinicaltrials.gov with registration ID NCT04767087.

**Full protocol:**

The full protocol is attached as an additional file, accessible from the Trials website (Additional file 1). With the intention of expediting dissemination of this trial, the conventional formatting has been eliminated; this Letter serves as a summary of the key elements of the full protocol. The study protocol has been reported in accordance with the Standard Protocol Items: Recommendations for Clinical Interventional Trials (SPIRIT) guidelines.

**Supplementary Information:**

The online version contains supplementary material available at 10.1186/s13063-021-05510-3.

## Supplementary Information

**Additional file 1: Full protocol.**

## Data Availability

All required data will be posted along with the trial results and final trail dataset requests can be made to Dr. Sohaib Ashraf which could be available from the author on reasonable appeal subject to data protection regulations. (Twitter: SohaibAshrafMD, email address: sohaib@skzmdc.edu.pk, Mobile Number: +92 3334474523)

