# Supplementary material for: Prophylactic potential of honey and Nigella sativa L. against hospital and community-based SARS-CoV-2 spread: a structured summary of a study protocol for a randomised controlled trial
Source: Trials. 2021 Sep 15;22:618. doi: 10.1186/s13063-021-05510-3 (PMC8441040; doi:10.1186/s13063-021-05510-3)
Supplement: Supplementary file 1 — Additional file 1: Full protocol. [file 13063_2021_5510_MOESM1_ESM.pdf]

Title

Honey and *Nigella sativa* in the prophylaxis of COVID-19; A randomized controlled trial

Trial Name

HNS-COVID-PK-II

Study Principal Investigators:

1. **Prof. Dr. Muhammad Ashraf, PhD (University of Minnesota)**
   Designation: Professor Emeritus of Pharmacology & Toxicology

   Institutional Affiliation: University of Veterinary & Animal Sciences, Lahore, Pakistan
2. **Dr. Shoaib Ashraf, PhD (McGill University)**
   Designation: Research Associate Parasitology & Pharmacology

   Institutional Affiliation: Massachusetts Hospital Harvard University, Boston, USA
3. **Dr. Moneeb Ashraf, M.Phil. (Federal Post-Graduate Medical Institute)**
   Designation: Associate Professor Pharmacology

   Institutional Affiliation: Mayo Hospital, Kingedward Medical University, Lahore, Pakistan
4. **Dr. Sohaib Ashraf, MBBS (Shaikh Khalifa Bin Zayed Al-Nahyan Medical & Dental College)**
   Designation: MD Post-Graduate Cardiology trainee

   Institutional Affiliation: Shaikh Zayed Post-Grad Medical Complex, Lahore, Pakistan

Co-Investigators:

1. **Prof. Dr. Ali Ahmad, PhD (Virology)**
   Institutional Affiliation: University of Montreal, Sainte Justin Hospital, Canada.
2. **Dr. Talha Mahmood, MD (Pulmonology)**
   Institutional Affiliation: Shaikh Zayed Medical Complex, Lahore, Pakistan
3. **Dr. Uzma Mamoon, FCPS(Medicine)**
   Institutional Affiliation: Shaikh Zayed Medical Complex, Lahore, Pakistan
4. **Dr Nighat Masood, FCPS (Medicine)**
   Institutional Affiliation: Services Institute of Medical Sciences, Lahore, Pakistan.
5. **Dr. Muhammad Ahmad Imran, MBBS**
   Institutional Affiliation: Microbiology Department, Shaikh Zayed Medical Complex, Lahore, Pakistan

Chief Site Investigators:

1. **Dr Larab Kalsoom, MBBS**
   Institutional Affiliation: Services Institute of Medical Sciences, Lahore, Pakistan
2. **Dr. Sundas Rafique, MBBS**
   Institutional Affiliation: Mayo Hospital, Kingedward Medical University, Lahore, Pakistan
3. **Dr. Iqra Farooq, MBBS**
   Institutional Affiliation: Children Hospital, Lahore, Pakistan
4. **Dr. Abeer Bin Awais, MBBS**
   Institutional Affiliation: Shaikh Zayed Hospital, Lahore, Pakistan

LEAD BIOSTATITICIAN:

Prof. Dr. Muhammad Azam, PhD

Institutional Affiliation: University of Veterinary & Animal Sciences, Pakistan.

RESEARCH PHARMACISTS & DRUG SAFETY PANELISTS:

1. **Dr. Asad-ullah, PharmD, Ph.D.**
   Institutional Affiliation: Drug Regulation Aurthority Pakistan (DRAP)
2. **Dr Zaka-ur-Rehman, PharmD, Ph.D.**
   Institutional Affiliation: Ministry of Health Punjab, Pakistan
3. **Dr. Syed Muhammad Muneeb, PharmD, PhD**
   Designation: Assistant Professor of Pharmaceutical Sciences
   Institutional Affiliation: Institute of Pharmaceutical Sciences, UVAS
4. **Dr. Faisal Nadeem, PharmD, PhD**
   Designation: Assistant Professor of Pharmaceutical Sciences

   Institutional Affiliation: Institute of Pharmaceutical Sciences, UVAS

CLINICAL EXPERT PANEL:

- **Dr. Talha Mahmood, MD (Pulmonology)**
  Institutional Affiliation: Shaikh Zayed Medical Complex, Lahore, Pakistan.
- **Dr. Qazi Abdul Saboor, FCPS(Cardiology)**
  Institutional Affiliation: Shaikh Zayed Medical Complex, Lahore, Pakistan.

TRIAL STEERING COMMITTEE

- **Prof. Dr. Mateen Izhar, PhD. (Chairman)**
  Institutional Affiliation: Shaikh Zayed Medical Complex, Lahore, Pakistan.
- **Prof. Dr. Xin Zhao, PhD.**
  Institutional Affiliation: McGill University, Sainte-Anne-de-Bellevue, Quebec, Canada
- **Prof. Dr. Ali Ahmad, PhD.**
  Institutional Affiliation: University of Montreal, Sainte Justin Hospital, Canada.
- **Prof. Dr. Amber Malik, MRCP.**
  Institutional Affiliation: Evercare Hospital, Lahore, Pakistan
- **Prof. Dr. Muhammad Azam, PhD.**
  Institutional Affiliation: University of Veterinary & Animal Sciences, Pakistan
- **Prof. Dr. Muhammad Imran, PhD**
  Institutional Affiliation: Massachusetts Institute of Technology, Boston, USA
- **Prof. Dr. Yasir Zahoor, PhD**
  Institutional Affiliation: Boston Children Hospital, Harvard Medical School, USA
- **Prof. Dr. Umer Naveed, PhD**
  Institutional Affiliation: Royal (Dick) School of Veterinary Studies and Roslin Institute, Scotland, UK
- **Prof. Dr. Fazli Mabood, PhD**
  Institutional Affiliation: CHU Sainte-Justine Hospital, University of Montreal, Canada
- **Prof. Dr. Muhammad Bilal, M.Phil.**
  Institutional Affiliation: McGill University, Sainte-Anne-de-Bellevue, Quebec, Canada
- **Syed Kazim Ali, LLB**
  Institutional Affiliation: Lahore High Court Bar, Pakistan

ABBREVIATIONS:

ARDS	Acute Respiratory Distress Syndrome
CBC	Complete Blood Count
CMV	Cytomegalovirus
COVID-19	Corona Virus Disease 19
DM	Diabetes Mellitus
FAIR	Findable, Accessible, Interoperable, Re-usable
HIV	*Human Immunodeficiency Virus*
HSV	Herpes Simplex Virus
HTN	Hypertension
IRB	Institutional Review Board
MRSA	Methicillin-resistant Staphylococcus aureus
NICE	National Institute For Health And Care Excellence
RCT	Randomized Control Trial
RFTs	Renal Function Tests
RT-PCR	Real Time Polymerase Chain Reaction
SARS-CoV-2	Severe acute respiratory syndrome coronavirus 2
SIMS	Services Institute of Medical Sciences
SZH	Shaikh Zayed Medical Complex
TQ	Thymoquinone
WHO	World Health Organization
$ZnCl_2$	Zinc Chloride

Administrative information

Title	Randomized, controlled, multi-centered, close-label, interventional study designed to demonstrate the prophylactic role of honey and Nigella sativa in the post-exposure COVID-19 patients.
Trial registration	Clinical Trial registration will be done at www.clinicaltrials.gov
Protocol version	Version 1.4 dated 12/24/2020
Sponsoring Body	Smile Welfare Organization
Author details {5a}	Dr Sohaib Ashraf<br>Post-Graduate Cardiology Resident, Federal Post-Graduate Medical Institute, Lahore, Pakistan.
Name and contact information for the trial sponsor {5b}	Syed Kazam Ali, kazimformanite@gmail.com
Role of sponsor {5c}	There will not be any influential role of sponsor/funder in implication of trial, the study design, analysis of data and interpretation of final result outcomes.

INTRODUCTION

Background and rationale {6a}

Sudden appearances of cluster pneumonia cases started in December 2019in Wuhan, China which was diagnosed to be the cause of SARS-CoV-2. Over time, the disease named as COVID-19 rapidly travelled to the entire world(Wang, Wang et al. 2020, Xie, Zhong et al. 2020). The COVID-19 epidemic was declared as a global pandemic on March 12, 2020 by WHO(Ayenew and Pandey 2020). Researchers throughout the world took the notice of proceedings and started exploring different facets of the disease. In context of its severity, a recent study illustrates that 80% of the positive tested participants present with mild disease symptoms and the overall mortality lies around 2.3%. However, in participants aged 70 to 79 years, this ratio is considerably higher (8.0%) while it reaches to 14.8% in participants aged >80 years(Wu and McGoogan 2020). It is estimated that majority of cases are disregarded due to either asymptomatic nature, lack of testing facilities or social dilemmas. What requires our immediate attention is the pursuit of an effective treatment for symptomatic participants. On the same lines, an intervention should also be sought to decrease viral latency duration of its shedding in order to overcome transmission in the community. In response to this emergency, a number of recent studies have been conducted to explore a potential miracle drug. Such efforts included novel antivirals like remdesivir and favipiravir on one side while testing many existing antiviral drugs like lopinavir/ritonavir, darunavir/umifenovir and ivermectin on the other side(Cai, Yang et al. 2020, Cao, Wang et al. 2020, Chaccour, Hammann et al. 2020, Chen, Xia et al. 2020, Grein, Ohmagari et al. 2020, Patel and Desai 2020, Wang, Zhang et al. 2020). Among the main highlights of such findings is hydroxychloroquine, which has shown promising anti-viral effects against SARS-CoV-2(Ferner and Aronson 2020, Gautret, Lagier et al. 2020, Geleris, Sun et al. 2020). With lesser media reporting, other studies have also suggested a positive impact of iron chelators and $ZnCl_2$ as well, in combating COVID-19. In parallel to these lines, many traditional therapies are also being interrogated for their possible antiviral properties against COVID-19(Te Velthuis, van den Worm et al. 2010, Contini 2020). Herbal extracts, evidence-based medicine, traditional therapeutics and therapies from folklores have continuously been put to rigorous scientific investigations in the past. One of the most studied amid traditional herbs is *Nigella sativa*. Lately, the widespread therapeutic effects of *Nigella sativa* seeds against many ailments have been historically documented and have been tested by researchers. The broad spectrum of ailments for *Nigella sativa* treatment include dermatological problems, gastrointestinal diseases, ophthalmological issues, autoimmune diseases, respiratory tract infections, gynecological problems, diabetes and

hypertension(Forouzanfar, Bazzaz et al. 2014). Till now, PubMed indexing reports more than 50 human clinical trials that provide a good rationale about its efficacy and safety. After fractionation, the most active constituent, Thymoquinone (TQ) has been attributed mainly for its activities. TQ has also been demonstrated to possess anticonvulsant(Hosseinzadeh, Parvardeh et al. 2005, Parvardeh, Nassiri-Asl et al. 2005), antioxidant(Zaher, Bendary et al. 2019), anti-inflammatory(El Gazzar, El Mezayen et al. 2006) and anti-cancer(Gali-Muhtasib, Ocker et al. 2008)properties. What has been interesting for us is its significant antimicrobial value which has shown promising results against Gram-positive and Gram-negative bacteria, parasites, viruses, fungi and Schistosoma(Forouzanfar, Bazzaz et al. 2014)

In milieu of our trial, it is quite encouraging that *Nigella Sativa* has been shown previously to inhibit viral replication, demonstrated antiviral effects against CMV (Salem and Hossain 2000), hepatitis C virus(Salem and Hossain 2000, Barakat, El Wakeel et al. 2013) and a number of other viruses such as HIV(Onifade, Jewell et al. 2015), H9N2(Forouzanfar, Bazzaz et al. 2014, Umar, Munir et al. 2016), mosaic virus and new castle disease virus(Forouzanfar, Bazzaz et al. 2014, Shamim Molla, Azad et al. 2019). We have quite encouraging findings which display that *Nigella sativa's* main ingredient, TQ has been shown to decrease viral replication in coronavirus-infected cells (SARS-CoV-1)(Ulasli, Gurses et al. 2014). Moreover, the reports of naturally occurring iron chelators and zinc in Nigella Sativa(Al-Jassir 1992, Takruri and Dameh 1998) which are already under exploration in COVID-19, additionally may contribute to its antiviral effects against SARS-CoV-2.

The second ingredient of our Anti-Covid-19 mix is natural honey. From the last six eras, human civilization records demonstrate the use of honey as medicine. Previous scrolls (1900–1250 BC) evidently reveal the effectiveness of honey as it was being used by different philosophers and physicians as a remedy for various illnesses(Khan, Anjum et al. 2018). Clinical data indicates that honey possesses strong anti-tussive, anti-cancerous (bladder cancer) and wound healing characteristics (Manuka Honey). Although traditionally believed from a long time, a study has confirmed the reproductive system benefits of honey including increases in concentration of serum testosterone, enhancements of sperm count and increased chances of conception(Khan, Anjum et al. 2018). Moreover, it also acts as cardio-protective(Onyesom 2004) , exhibits anti-diabetic(Erejuwa, Sulaiman et al. 2012, Bobiş, Dezmirean et al. 2018) and laxative properties. Our interest in honey revolves around its strong antimicrobial efficacy. Despite being a food in nature, honey is immune to microbial attacks. In the 1960's with the emergence of highly efficient antibiotics, honey was comparatively perceived as a "worthless

but harmless substance”(Soffer 1976). However, a recent burst in emergence of antibiotic resistance where we lack remedies against highly resistant microbes, attentiveness towards honey is being resuscitated. Many studies have optimized the use of honey as an effective antimicrobial agent and as a therapeutic drug for various infections. As honey has been reported by scientific annals to possess anti-bacterial, anti-viral, anti-oxidative and anti-inflammatory properties, modern research has verified the therapeutic inevitability of honey. Honey is bactericidal. A strong anti-bacterial activity against Gram-positive bacteria (B.subtilis,S.aureus, and Paenibacillus larvae)(Kwakman and Zaat 2012) and Gram-negative bacteria (Pseudomonas aeruginosa, Enterobacter spp., Klebsiella) has been demonstrated in different studies(Abd-El Aal, El-Hadidy et al. 2007). Furthermore, in cases of highly resistant Gram-positive methicillin-resistant *Staphylococcus aureus,* a 100% inhibition was detected(Almasaudi, Al-Nahari et al. 2017). Such antimicrobial effects of Honey could be attributed towards its sugar concentration, its unique constituents and elevated pH values(Khan, Anjum et al. 2018). Further studies show synergistic effects of honey with antibiotic agents comparable to oxacillin, tetracycline, imipenem and meropenem against MRSA(Jenkins and Cooper 2012). In perspective of its antiviral activity, as hypothesized in our trials, previously it has demonstrated antiviral effects against rubella virus, herpes simplex virus (HSV), hepatitis and anti-Varicella Zoster virus(Khan, Anjum et al. 2018). A stimulating aspect of currently tested anti-covid-19 therapies could be the common trait of being acidic in nature. The recent therapies tested as antiviral drugs for COVID-19 are remdesivir, lopinavir and ritonavir which are organic acids, like honey. Another aspect of a prominent similarity is that lopinavir gives phenolic dimers(RAGHAVA REDDY, Garaga et al. 2015) and both of these (organic and phenolic) properties are also shown by honey(da Silva, Gauche et al. 2016).

In addition to its direct antiviral effects, honey also exhibits immunity-booster effects mainly via polyphenolic components. The proliferation of lymphocytes (B and T), along with the production of antibodies during primary and secondary immune response is stimulated via honey’s phenolic ingredients(Boukraâ 2013). Honey is known to activate the immune response via stimulation of human monocytic cells to release inflammatory cytokines (e.g. TNF-a, IL-1and IL-6)(Boukraâ 2013). Taken together, honey could be a vastly effective therapeutic agent against COVID-19 either through its antiviral properties, its immune boosting characteristics or the blend of both.

While considering its safety profile in COVID-19 participants, we have found that more than 250 human clinical trials have been conducted to determine efficacy and safety profiles of honey in different ailments. As an outcome, NICE and Public Heath England guidelines

recommend honey as the first line of treatment for an acute cough caused by upper respiratory tract infections, known as one of the defining symptoms of COVID-19.

An Erythromycin derivative, Azithromycin, shows improved activity against Gram-negative bacteria. It causes premature chain termination during translation via inhibitory effect in Escherichia coli, in which a peptide bond is formed between puromycin tRNA bound at the P-site of poly(U)-programmed ribosomes(Dinos, Michelinaki et al. 2001) Puromycin which is an aminoglycoside antibiotic is also found in Honey and it shows aminoglycoside effect against pseudomonas aeruginosa even better than gentamycin and amikacin(Chauhan and Desai 2013)

We believe that we have surplus data regarding proposed activities, pharmacokinetics and safety profiles in favor of our proposed therapeutic regimen. Therefore, we have planned to conduct a clinical trial aiming at the observation of combined effect of *Nigella sativa* and honey on SARS-CoV-2-infected participants. The aim of the study is to explore the possible Prophylactic use of *Nigella sativa* and honey in the treatment of Corona virus infection. This effect has not been investigated in earlier human trials.

Objectives (7)

The objective of the study is to measure the efficacy of Honey and *Nigella sativa* as a prophylactic agent in experimental group as compared to control group.

Trial design (8)

This is an adaptive, multi-centered design with 1:1 allocation ratio and superiority framework.

Methods: Participants, interventions and outcomes

Study setting (9)

The study centers chosen for this clinical trial will include:

- Shaikh Zayed Medical Complex, Lahore, Pakistan
- Ali Clinic, Lahore, Pakistan.
- Doctors' Lounge, Pakistan.

Eligibility criteria (10)

Asymptomatic participants (health care workers and households) presenting within 4 days of exposure to a confirmed COVID-19 case will be screened.

<u>Inclusion Criteria:</u>

- Both genders and age 18 years and above
- 4 days of exposure to a confirmed COVID-19 case

<u>Exclusion Criteria:</u>

- Presence of co-morbidities like liver disease, immuno-compromised participant or any other chronic ailment (other than Diabetes and Hypertension)
- Females who are pregnant and breast feeding
- Uncontrolled Diabetes Mellitus and Hypertension
- If a participant is allergic to either honey or *Nigella sativa*.
- Refusal or withdrawal from informed consent.
- Participant with COVID-19 symptoms
- Participant with positive COVID-19
- Hemodynamic instability

Who will take informed consent? (26a)

Site investigator will take written informed consent from all trial participants by giving them the specifically constructed informed consent form (provided at the end of proposal)

Additional consent provisions for collection and use of participant data and biological specimens {26b}

Site investigators will also be responsible for taking consent regarding every other matter if required. Informed consent form will contain section on permission to draw and conduct specified tests on blood samples and conduct radiological investigations as per protocol of the study.

Interventions

Explanation for the choice of comparators (6b)

We will have two arms, experimental and the control arm. The experimental arm will be receiving honey and nigella *sativa* to see the prophylaxis effect for 14 days. Whereas the control arm would be receiving nothing at all.

Intervention Description (11a)

High quality honey and *Nigella sativa* seeds will be provided by smile welfare organization, Lahore, Pakistan. Both products will be certified by botanical department of Government College University, Lahore Pakistan. *Nigella sativa* seeds (40 mg/kg/day) will be grinded and packed in capsules which will be given to the interventional group along with 0.5 gm/kg/day of honey stirred in 250ml of warm water in 2 to 3 divided doses, for 12 weeks.

Criteria for discontinuing or modifying allocated interventions (11b)

Fixed dose will be given throughout the study and interventional drug administration will be stopped immediately in following conditions:

1. Any adverse drug reaction
2. Participant denies/back off from the further participation
3. Participants gets COVID-19 positive

Regardless of any of the condition, participant's data will be retained and analyzed in the trial in order to follow-up and prevent any data missing.

Strategies to improve adherence to interventions (11c)

To improve adherence to the intervention, participants will be counselled about the advantages of this study. All the participants will be monitored regarding compliance to their assigned treatment strategy and site investigators will give drugs. Weekly structured telephones calls will be made to ensure compliance and adherence to the interventions. In case of unsuccessful phone calls, site investigators will be asked for additional information including phone numbers of close relatives, e-mail addresses, and attempted again to contact the participant or a relative. If these attempts would go unsuccessful, as a last resort, for participants living in Lahore, a trained person will visit the subjects house and obtain follow-up data. Distribution of honey, *Nigella sativa* will be the responsibility of Smile Welfare Organization.

Relevant concomitant care permitted or prohibited during the trial (11d)

As per hospital protocol (study setting), the care and interventions permitted will be used and no specific prohibited care is in this trial.

Provisions for post-trial care (30)

No specific post-trial care will be needed as no sound documented scientific evidence is there about adverse effects with Honey or *Nigella sativa* with this dose for this trial.

Recruitment criteria (15):

Health care workers, in above mentioned study settings, who have not contacted COVID-19 will be recruited via site investigators.

OUTCOMES (12)

Primary Outcomes:

- Incidence of COVID-19 cases or SARS CoV-2 infection rate i.e. number of confirmed participants within 14 days

Secondary Outcomes:

- Incidence of COVID-19-related symptoms, hospitalizations and deaths
- The severity of COVID-19-related symptoms

Participant timeline {13}

Time schedule of enrolment, interventions (including any run-ins and washouts), assessments, and visits for participants has been shown in a schematic diagram.

Sample size and Study Duration (14)

An initial sample size of 1000 participants, 500 participants in each arm will be taken for this multi-centered study in Pakistan. An inter rim analysis will be done when half of the patients have been recruited to evaluate the need to adapt sample size, efficacy, and futility of the trial.

Recruitment (15)

Recruitment will be done from Services Institute of Medical Sciences and Shaikh Zayed Medical Complex. Eligible participants will be identified using our eligibility criteria (mentioned above). Participants will be stratified into high-risk exposure (participant being at a distance of less than 6 feet for more than 10 minutes while wearing neither a face mask nor an eye shield) or moderate-risk exposure (participant being at a distance of less than 6 feet for more than 10 minutes while wearing a face mask but no other personal protective equipment). Participants will be recruited in each arm after taking complete written informed consent by the site investigators.

Assignment of interventions: allocation

Sequence generation (16a)

Stratification will be done via SAS software version 9.4 for initial COVID-19 on risk exposure to ensure that groups remain balanced in size for either arm after written informed consent to participate in our study. Randomization will be done using a lottery method. As participants might be admitted at different times, they will be recruited after taking written informed consent (following all standard protocol for infection control and disinfection). To minimize allocation bias, we will perform allocation concealment with an interactive voice/web-based response system until randomization will be finished on the system through a computer or phone.

Concealment mechanism (16b)

The allocation sequence will be computer generated that will be concealed from all site investigators, allocated participants and treatment providers until the final interventional allocation is done.

Implementation (16c)

The site investigators who will do the recruitment of interventional groups will request the principal investigator, Shoaib Ashraf (ShA) for randomization. The principal investigator (ShA) will send his answer form to the treatment providers in concealed envelops at allocated corona centres. The therapists will have no influential role in study outcomes and analysis while only disclosing the treatment plans to patients. Site investigators and other study members that are involved in participant's enrolment will not be allowed to receive allocation information in order to prevent study bias.

Assignment of interventions: Blinding

Who will be blinded? (17a)

Outcome assessors and data analysts are blinded, respectively, by using site investigators to provide drugs to participants while by using statistical analysts from other institutions that are not having any conflict of interest in research.

Procedure for unblinding if needed (17b)

Unblinding is permissible if the participants develop COVID-19 symptoms. If unblinding is required, the trial managers and data coordinators will have access to group allocations and any unblinding will be reported.

Data collection and management (18a)

Microsoft Excel will be used to ensure the data safety. Two site investigators will enter the data, recheck twice for possible errors separately, and make certain its integrity. There is no conflict of financial and non-financial interest with sponsors and researchers.

Plans to promote participant retention and complete follow-up (18b)

Most of the data will be collected while the subjects are working in their respective centers and follow up will be done using phone numbers of the subjects.

Data management (19)

Participants IDs will be used for confidentiality purposes and these IDs will be linked to demographic information securely and separately. The final data set of RCT will have coded data and can only be assessed by principal investigators. All outcomes will be double checked by the researchers prior to data collection and data storage. To ensure data's integrity and safety various meetings by the research team will be conducted

Confidentiality (27)

In addition, confidentiality of participants' data will be ensured by using participants' IDs rather than identifiable information in the dataset (i.e. coding) and personal data will be optional for the participants considering their regional and religious believes though storing the document linking the IDs to the identifiable information separately and securely.

Statistical methods

Statistical methods for primary and secondary outcomes (20a)

Biostatistician using SAS version 9.4 will analyse all trial data. Mean ± S.D will be used for quantitative data and f (%) will be used for categorical data. Frequency and percentages will be measured for categorical data. Data normality will be checked using Shapiro Wilks test, if data is normal independent sample t-test will be used to compare quantitative outcome. Mann Whitney U test will be used to compare median of these quantitative data. Chi-square t-test. For follow up analysis Wilcoxon test will be applied. If data supports the necessary assumptions of time to event data, survival analysis/ Kaplan Meier test will be applied. P-value < 0.05 will be considered as significant.

Interim analyses (21b)

The risk aptitude for this study is customized as mild risk so one interim analysis is planned at recruitment of total 500 participants followed by sample size re-estimation.

Methods for additional analyses (e.g. subgroup analyses) (20b)

Adjusted and subgroups analysis may be applied as per the biostatistician, if needed. In that case both unadjusted and adjusted analyses are provided along with the main analysis

Methods in analysis to handle protocol non-adherence and any statistical methods to handle missing data (20c)

The intention-to-treat analysis set will be used to test the superiority. All patients will be considered as randomized despite receiving the randomized treatment as per our anticipation. Reasons for each group's randomization and withdrawal will be reported and compared qualitatively and sensitivity analysis (augmented data) is being used to overcome the effect of any missing data on results. The participants who withdraw consent for continued follow-up (Dropouts) will be assessed by modern imputation methods for missing data

Plans to give access to the full protocol, participant level-data and statistical code (31c)

Principal investigators will have access to the full trial dataset in order to ensure that the overall results are not disclosed by an individual of trial prior to the main publication. Grant public access to the full protocol, participant-level dataset, and statistical code will be given through clinicaltrials.gov.

Oversight and monitoring

Composition of the coordinating center and trial steering committee (5d)

Coordinating centre and trial executive committee will be comprised of medical lab technologists, biochemists, clinical pharmacists, clinical pharmacologists and toxicologists, virologists, immunologists, biostatisticians, public health experts, epidemiologists, ethical experts and consultants of medicine, pulmonology, cardiology and infectious disease departments. This committee will be responsible for the safety and results of endpoints. This will have all the authority to stop the clinical trial all together. Site investigators will be responsible for data collection and quality check of data at collection points/ study setting.

Composition of the data monitoring committee, its role and reporting structure (21a)

The executive committee vouches for the completeness and accuracy of the data and for the fidelity of the trial to the protocol.

Data Collection:

Data will be collected daily, from randomization until 14 days, in the case-report form. Data will be collected directly via structured telephones calls as virtual visits on day 1, 5 and 14.

Adverse event reporting and harms (22)

The researchers will record any adverse, unpredictable or undesirable sign and symptom and it will be discussed with the care providers. A comprehensive evaluation will be conducted to evaluate the co relation between experimental drug and the developing signs and symptoms. The investigator will respond appropriately to ensure the wellbeing of the participant in case of any unforeseen event and all the details will be written carefully. Moreover, regular follow-ups will be made certain until the participant regains his/her health. If the adverse event happens during the study intervention will be reported to Institutional Review Board (IRB).

Frequency and plans for auditing trial conduct (23)

Auditing of the clinical trial will be done by trial steering committee in meetings where all the audits will be provided by the site investigators and research coordinators.

Plans for communicating important protocol amendments to relevant parties (e.g. trial participants, ethical committees) (25)

In order to modify protocols including eligibility criteria and outcomes, permission will be needed to get approved by the trial steering committee and the notification will be done to relevant parties including IRB trial registries and journals.

Dissemination plans (31a)

The publications subcommittee will review the publication, all the endpoint data, primary outcome analysis and the study results and recommend the changes to the author. After the changes being done it will finally submit its recommendations to the steering committee for approval. Study results will be disclosed to all study participants, member physicians, participants and other medical personnel.

Availability of data and materials (29)

The datasets used or analysed during the current study will be available from the corresponding author upon reasonable requests.

Ethics approval and consent to participate {24}

Ethical approval will be obtained from institutional review board of Shaikh Zayed Hospital, Lahore, PK. Written, informed consent to participate will be obtained from all participants.

Competing interests (28)

The authors affirm that they have no competing interests and according to the standards of scientific integrity the publication of both positive and negative study results will be ensured. All the study data (if reasonable) will be made accessible guided by the FAIR principles along with the perspective of relevant laws and privacy regulations. Authorship eligibility follows conventional academic standards. No professional writers have been involved in this.

Funding: (4)

Smile Welfare Organization

Figure 1: Enrolment Criteria:

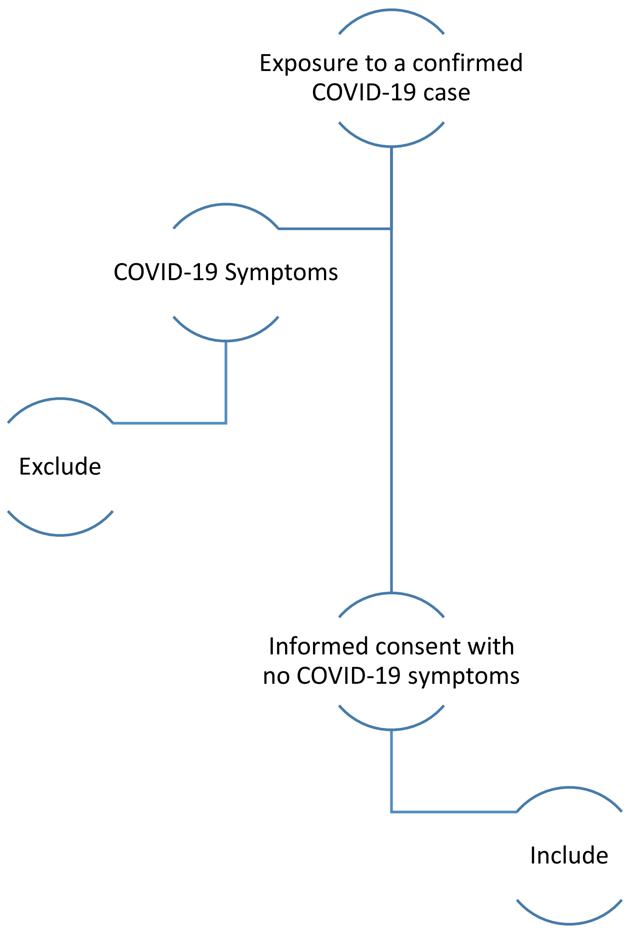

CONSORT 2010 Flow Diagram

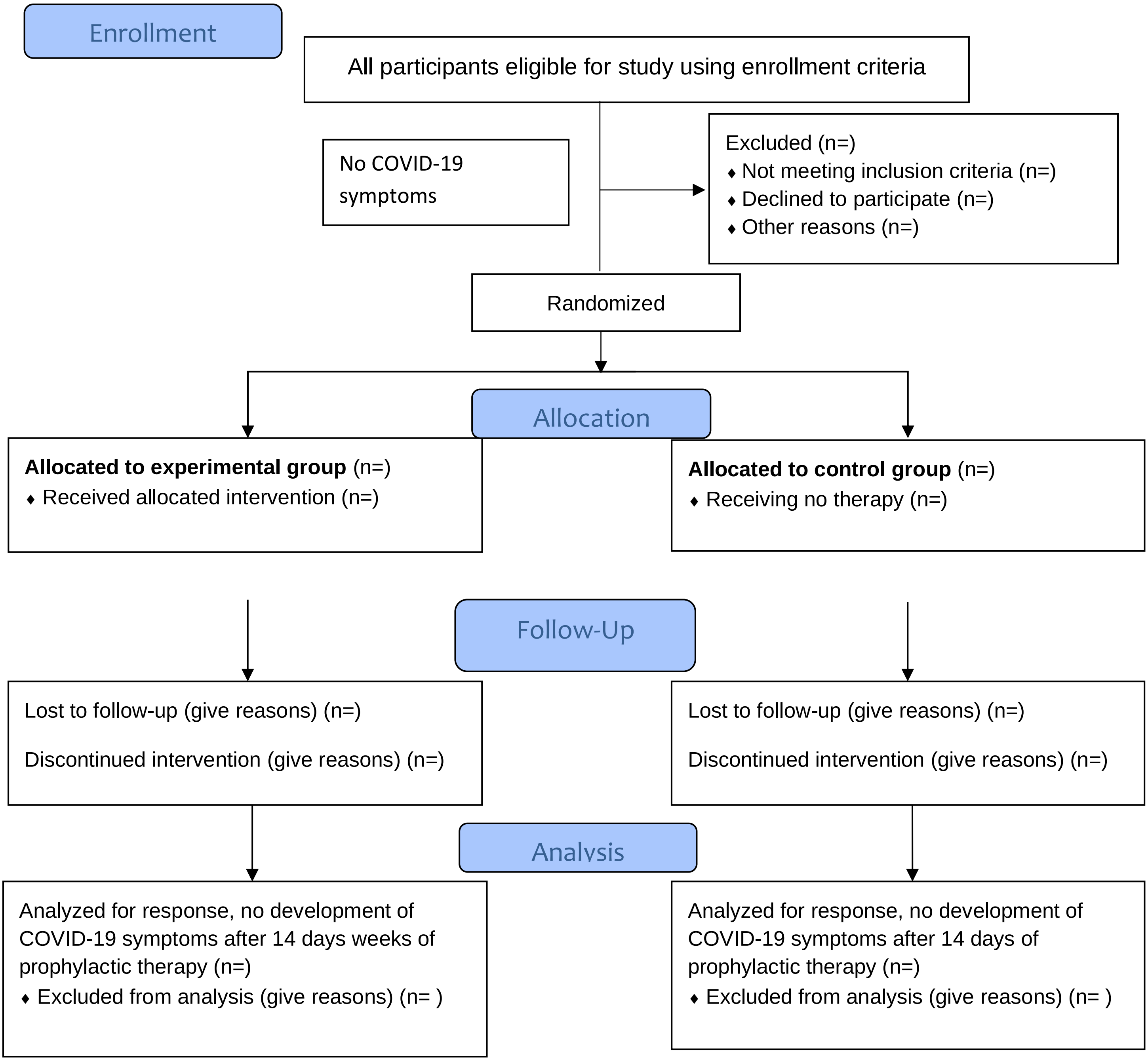

References:

Abd-El Aal, A., M. El-Hadidy, N. El-Mashad and A. El-Sebaie (2007). "Antimicrobial effect of bee honey in comparison to antibiotics on organisms isolated from infected burns." Annals of Burns and fire disasters **20**(2): 83.

Al-Jassir, M. S. (1992). "Chemical composition and microflora of black cumin (Nigella sativa L.) seeds growing in Saudi Arabia." Food Chemistry **45**(4): 239-242.

Almasaudi, S. B., A. A. Al-Nahari, M. El Sayed, E. Barbour, S. M. Al Muhayawi, S. Al-Jaouni, E. Azhar, M. Qari, Y. A. Qari and S. Harakeh (2017). "Antimicrobial effect of different types of honey on Staphylococcus aureus." Saudi journal of biological sciences **24**(6): 1255-1261.

Amit N. Patel MD, M., 2; Sapan S. Desai MD PhD MBA3; David W. Grainger PhD1; Mandeep R. Mehra,MD, MSc4 "Usefulness of Ivermectin in COVID-19 Illness."

Ayenew, B. and D. Pandey (2020). "Challenges and opportunities to tackle COVID-19 spread in Ethiopia." Journal of PeerScientist **2**(2): e1000014.

Barakat, E. M. F., L. M. El Wakeel and R. S. Hagag (2013). "Effects of Nigella sativa on outcome of hepatitis C in Egypt." World journal of gastroenterology: WJG **19**(16): 2529.

Bobiş, O., D. S. Dezmirean and A. R. Moise (2018). "Honey and diabetes: the importance of natural simple sugars in diet for preventing and treating different type of diabetes." Oxidative Medicine and Cellular Longevity **2018**.

Boukraâ, L. (2013). Honey in traditional and modern medicine, CRC Press.

Cai, Q., M. Yang, D. Liu, J. Chen, D. Shu, J. Xia, X. Liao, Y. Gu, Q. Cai and Y. Yang (2020). "Experimental treatment with favipiravir for COVID-19: an open-label control study." Engineering.

Cao, B., Y. Wang, D. Wen, W. Liu, J. Wang, G. Fan, L. Ruan, B. Song, Y. Cai and M. Wei (2020). "A trial of lopinavir–ritonavir in adults hospitalized with severe Covid-19." New England Journal of Medicine.

Chaccour, C., F. Hammann, S. Ramón-García and N. R. Rabinovich (2020). "Ivermectin and COVID-19: Keeping Rigor in Times of Urgency." The American Journal of Tropical Medicine and Hygiene **102**(6): 1156.

Chauhan, P. and P. Desai (2013). "Antipseudomonal property of honey and aminoglycosides." Journal of Applied and Natural Science **5**(2): 338-341.

Chen, J., L. Xia, L. Liu, Q. Xu, Y. Ling, D. Huang, W. Huang, S. Song, S. Xu and Y. Shen (2020). Antiviral Activity and Safety of Darunavir/Cobicistat for the Treatment of COVID-19. Open forum infectious diseases, Oxford University Press US.

Contini, A. (2020). "Virtual screening of an FDA approved drugs database on two COVID-19 coronavirus proteins."

da Silva, P. M., C. Gauche, L. V. Gonzaga, A. C. O. Costa and R. Fett (2016). "Honey: Chemical composition, stability and authenticity." Food chemistry **196**: 309-323.

Dinos, G. P., M. Michelinaki and D. L. Kalpaxis (2001). "Insights into the Mechanism of Azithromycin Interaction with anEscherichia coli Functional Ribosomal Complex." Molecular pharmacology **59**(6): 1441-1445.

El Gazzar, M., R. El Mezayen, J. C. Marecki, M. R. Nicolls, A. Canastar and S. C. Dreskin (2006). "Anti-inflammatory effect of thymoquinone in a mouse model of allergic lung inflammation." International immunopharmacology **6**(7): 1135-1142.

Erejuwa, O. O., S. A. Sulaiman and M. S. Ab Wahab (2012). "Honey-a novel antidiabetic agent." International Journal of Biological Sciences **8**(6): 913.

Ferner, R. E. and J. K. Aronson (2020). Chloroquine and hydroxychloroquine in covid-19, British Medical Journal Publishing Group.

Forouzanfar, F., B. S. F. Bazzaz and H. Hosseinzadeh (2014). "Black cumin (Nigella sativa) and its constituent (thymoquinone): a review on antimicrobial effects." Iranian journal of basic medical sciences **17**(12): 929.

Gali‐Muhtasib, H., M. Ocker, D. Kuester, S. Krueger, Z. El‐Hajj, A. Diestel, M. Evert, N. El‐Najjar, B. Peters and A. Jurjus (2008). "Thymoquinone reduces mouse colon tumor cell invasion and inhibits

tumor growth in murine colon cancer models." Journal of cellular and molecular medicine **12**(1): 330-342.
Gautret, P., J.-C. Lagier, P. Parola, L. Meddeb, M. Mailhe, B. Doudier, J. Courjon, V. Giordanengo, V. E. Vieira and H. T. Dupont (2020). "Hydroxychloroquine and azithromycin as a treatment of COVID-19: results of an open-label non-randomized clinical trial." International journal of antimicrobial agents: 105949.
Geleris, J., Y. Sun, J. Platt, J. Zucker, M. Baldwin, G. Hripcsak, A. Labella, D. K. Manson, C. Kubin and R. G. Barr (2020). "Observational study of hydroxychloroquine in hospitalized patients with Covid-19." New England Journal of Medicine.
Gorbalenya, A. E., L. Enjuanes, J. Ziebuhr and E. J. Snijder (2006). "Nidovirales: evolving the largest RNA virus genome." Virus Res **117**(1): 17-37.
Grein, J., N. Ohmagari, D. Shin, G. Diaz, E. Asperges, A. Castagna, T. Feldt, G. Green, M. L. Green and F.-X. Lescure (2020). "Compassionate use of remdesivir for patients with severe Covid-19." New England Journal of Medicine **382**(24): 2327-2336.
Hosseinzadeh, H., S. Parvardeh, M. Nassiri-Asl and M.-T. Mansouri (2005). "Intracerebroventricular administration of thymoquinone, the major constituent of Nigella sativa seeds, suppresses epileptic seizures in rats." Medical Science Monitor **11**(4): BR106-BR110.
Jenkins, R. and R. Cooper (2012). "Improving antibiotic activity against wound pathogens with manuka honey in vitro." PloS one **7**(9): e45600.
Khan, S. U., S. I. Anjum, K. Rahman, M. J. Ansari, W. U. Khan, S. Kamal, B. Khattak, A. Muhammad and H. U. Khan (2018). "Honey: Single food stuff comprises many drugs." Saudi journal of biological sciences **25**(2): 320-325.
Kwakman, P. H. and S. A. Zaat (2012). "Antibacterial components of honey." IUBMB life **64**(1): 48-55.
Onifade, A. A., A. Jewell and A. Okesina (2015). "Seronegative conversion of an HIV positive subject treated with Nigella sativa and honey." African Journal of Infectious Diseases **9**(2): 47-50.
Onyesom, I. (2004). "Effect of Nigerian citrus (Citrus sinensis Osbeck) honey on ethanol metabolism." South African Medical Journal **94**(12).
Parvardeh, S., M. Nassiri-Asl, M. Mansouri and H. Hosseinzadeh (2005). "Study on the anticonvulsant activity of thymoquinone, the major constituent of Nigella sativa L. seeds, through intracerebroventricular injection." Journal of Medicinal Plants **2**(14): 45-52.
Patel, A. and S. Desai (2020). "Ivermectin in COVID-19 related critical illness." Available at SSRN 3570270.
RAGHAVA REDDY, A. V., S. Garaga, C. Takshinamoorthy and A. Naidu (2015). "Synthesis and Characterization of Impurities in the Production Process of Lopinavir." Scientia pharmaceutica **83**(1): 49-63.
Salem, M. L. and M. S. Hossain (2000). "Protective effect of black seed oil from Nigella sativa against murine cytomegalovirus infection." International journal of immunopharmacology **22**(9): 729-740.
Shamim Molla, M., A. K. Azad, M. A. A. Al Hasib, M. M. Hossain, M. S. Ahammed, S. Rana and M. T. Islam (2019). "A REVIEW ON ANTIVIRAL EFFECTS OF NIGELLA SATIVA L."
Soffer, A. (1976). "Chihuahuas and laetrile, chelation therapy, and honey from Boulder, Colo." Archives of Internal Medicine **136**(8): 865-866.
Takruri, H. R. and M. A. Dameh (1998). "Study of the nutritional value of black cumin seeds (Nigella sativaL)." Journal of the Science of Food and Agriculture **76**(3): 404-410.
Te Velthuis, A. J., S. H. van den Worm, A. C. Sims, R. S. Baric, E. J. Snijder and M. J. van Hemert (2010). "Zn2+ inhibits coronavirus and arterivirus RNA polymerase activity in vitro and zinc ionophores block the replication of these viruses in cell culture." PLoS pathogens **6**(11): e1001176.
Ulasli, M., S. A. Gurses, R. Bayraktar, O. Yumrutas, S. Oztuzcu, M. Igci, Y. Z. Igci, E. A. Cakmak and A. Arslan (2014). "The effects of Nigella sativa (Ns), Anthemis hyalina (Ah) and Citrus sinensis (Cs) extracts on the replication of coronavirus and the expression of TRP genes family." Molecular biology reports **41**(3): 1703-1711.

Umar, S., M. T. Munir, S. Subhan, T. Azam, Q. Nisa, M. I. Khan, W. Umar, Z. Rehman, A. S. Saqib and M. A. Shah (2016). "Protective and antiviral activities of Nigella sativa against avian influenza (H9N2) in turkeys." J. Saudi Soc. agric. Sci.
Wang, L.-s., Y.-r. Wang, D.-w. Ye and Q.-q. Liu (2020). "A review of the 2019 Novel Coronavirus (COVID-19) based on current evidence." International journal of antimicrobial agents: 105948.
Wang, Y., D. Zhang, G. Du, R. Du, J. Zhao, Y. Jin, S. Fu, L. Gao, Z. Cheng and Q. Lu (2020). "Remdesivir in adults with severe COVID-19: a randomised, double-blind, placebo-controlled, multicentre trial." The Lancet.
Wu, Z. and J. M. McGoogan (2020). "Characteristics of and important lessons from the coronavirus disease 2019 (COVID-19) outbreak in China: summary of a report of 72 314 cases from the Chinese Center for Disease Control and Prevention." Jama **323**(13): 1239-1242.
Xie, X., Z. Zhong, W. Zhao, C. Zheng, F. Wang and J. Liu (2020). "Chest CT for typical 2019-nCoV pneumonia: relationship to negative RT-PCR testing." Radiology: 200343-200343.
Zaher, M. F., M. A. Bendary, G. S. Abd El-Aziz and A. S. Ali (2019). "Potential Protective Role of Thymoquinone on Experimentally-induced Alzheimer Rats." Journal of Pharmaceutical Research International: 1-18.

CONSENT FORM (32)

This consent form is designed to give information regarding the trial and to obtain a well-informed consent of the enrolled participants.

Title: Effect of honey and *Nigella sativa* in the prophylaxis of COVID-19; A randomized controlled trial

Principal Investigator: Dr. Sohaib Ashraf

Name of Organization: Shaikh Zayed Medical Complex Lahore and Services Institute of Medical Sciences

The Consent Form is divided into two parts:

• Part 1, gives you a brief introduction about the trial

• Part 2, is the Consent Form

PART I:

Introduction:

I am ________________________ our team is conducting a research on prophylaxis against COVID-19, which is a global health concern. I am highlighting all the relevant information and inviting you to be part of this trail. Prior to any decision concerning your participation, take your time and talk to someone you feel at ease with about this research. If you have any difficulty understanding anything, you may stop me, and I will try my best to explain as I walk you through the information. If you still have any queries, you can clear them with the relevant doctor or the staff. A copy of this form will also be given to you.

Explanation:

The proposed study is a randomized controlled trial using parallel group designs. This is an open-label and adaptive, multi-centered design with 1:1 allocation ratio and superiority framework. This study will have two arms interventional arm will be Honey and *Nigella sativa* while other arm will be control arm receiving nothing. Data will be collected on self-constructed, close-ended questionnaires after obtaining written consent. Data will be analyzed using SAS version 9.4.

Primary outcome will be analyzed in both groups. The trial will aid in devising a better strategy to cope COVID-19 in a relatively inexpensive and accessible. The implications are global, and this could prove itself to be the most manageable intervention against COVID-19 especially for participants from limited-resource countries with deprived socioeconomic statuses.

Voluntary Participation:

It's a voluntary participation in this trial. Whether you choose to be a part of it or not, all the services you receive at this hospital will continue and nothing will change. If you choose to participate you will have the authority to change your mind later and stop participating even if you agreed earlier.

Procedures and Protocol

Participants in one group will be given the experimental treatment along while participants in the other group will only be given no drug at all. The healthcare workers will be monitoring you and the other volunteers' vigilantly during the study. If there is anything you are anxious about or that is troubling you about the research, please talk to me or one of the other colleagues.

You will not be monetarily benefited to take part in this research and confidentiality will be maintained. Identity of those taking part in this trial will not be revealed. The personal information that we collect from this research project will be kept confidential. Your personal information will be given numbers instead of your names. Only the researchers will be aware of the assigned number and we will lock that information up with a lock and key. This information can only be accessed by study director and study chair.

Sharing the Results:

Prior to making the knowledge, we get from this research, publically available to the outside world; it will be shared with the participants through community/zoom meetings. Confidential information will not be shared. After these meetings, we will publish the results so that the knowledge gained can be shared with rest of the world.

Right to Refuse or Withdraw:

You do not have to take part in this research if you do not wish to do so. You may also stop participating in the research at any time you choose. It is your choice and all of your rights will still be respected. Alternatives to Participating If you do not wish to take part in the research, you will be provided with the established standard treatment available at the center/institute/hospital.

Who to Contact:

If you have any questions you may ask them now or later, even after the study has started. If you wish to ask questions later, you may contact any of the following:

Dr Sohaib Ashraf +923334474523,

Dr Muhammad Ahmad Imran +923338110708

Dr Shahroze Arshad +923244516332

Do you have any questions?

PART II:

I have read the foregoing information, or it has been read to me. I have had the opportunity to ask questions about it and any questions that I have asked have been answered to my satisfaction. I consent voluntarily to participate as a participant in this research.

Print Name of Participant__________________

Signature of Participant ___________________

Date ___________________________ Day/month/year

If Illiterate (consent form being read to the witness) I have witnessed the accurate reading of the consent form to the potential participant, and the individual has had the opportunity to ask questions. I confirm that the individual has given consent freely.

Print name of witness_____________________

Thumb print of participant/ Signature of witness ______________________

Date ________________________ Day/month/year

I have accurately read out the information sheet to the potential participant, and to the best of my ability made sure that the participant understands. I confirm that the participant was given an opportunity to ask questions about the study, and all the questions asked by the participant have been answered correctly and to the best of my ability.

I confirm that the individual has not been coerced into giving consent, and the consent has been given freely and voluntarily.

A copy of this ICF has been provided to the participant.

Name of the person taking the consent ________________________

Signature of Researcher /person taking the consent__________________________

Date ___________________________ Day/month/year:

QUESTIONAIRE

Medical Record #: ______________________________

ID: ____

Age: ____

Gender: Male / Female

Exposure Level: ________________________________

Study Center: ___________________________________

Date of recruitment: ______________________________

Date of symptom development: ____________________

Contact Number: ________________________________

Profession: ______________________________________

Blood Group: ____________________________________

E-Mail: ____
